# A Method to Increase the Frequency Stability of a TCXO by Compensating Thermal Hysteresis

**DOI:** 10.3390/s20236812

**Published:** 2020-11-28

**Authors:** Zhaoyang Wang, Jie Wu

**Affiliations:** 1 Department of Modern Physics, University of Science and Technology of China, Hefei 230026, China; wangzy5@mail.ustc.edu.cn; 2 State Key Laboratory of Particle Detection and Electronics, University of Science and Technology of China, Hefei 230026, China

**Keywords:** TCXO, crystal oscillator, thermal hysteresis, digital compensation

## Abstract

Due to the rapid growth of electronic information technology, the need for the higher stability of crystal oscillators has increased. The temperature-compensated X’tal (crystal) oscillator (TCXO), a type of crystal oscillator with high frequency stability, has been widely used in communications, sensor networks, automotive electronics, industrial control, measuring devices, and other equipment. The traditional TCXO only performs frequency compensation based on the current temperature, without considering the error caused by thermal hysteresis. As the frequency stability of the TCXO improves, the thermal hysteresis of the crystal oscillator has a negligible influence on the frequency stability of the crystal oscillator. This study measured different compensation tables for hysteresis curves at different temperatures and used a microprocessor to store the historical information of crystal temperature changes. Furthermore, corresponding algorithms were designed to select the correct values, according to the temperature change history, to compensate for the thermal hysteresis of the crystal oscillator error. Experiments show that this method can reduce the hysteresis error of the crystal oscillator from 700 to 150 ppb (−40 to 80 °C).

## 1. Introduction

Clock signals are critical in numerous electronic systems, particularly wireless sensor networks and wireless Internet of Things devices that contain multiple sensors [1,2,3,4]. In these systems, multiple devices are often required to coordinate work, which involves clock synchronization between devices [5], and the clock stability of the crystal oscillator in each device is a key factor in determining the synchronization effect [6,7]. However, the frequency stability of general crystal oscillators is not high, and is mainly affected by environmental temperature [8]. Both the TCXO and the oven-controlled X’tal (crystal) oscillator (OCXO) can reduce the influence of crystal working environment temperature on their output frequency. The TCXO maintains the stability of the frequency by compensating the frequency offset of the crystal oscillator caused by temperature changes, whose stability is generally around 1 ppm. The OCXO keeps its output frequency stable by raising the temperature around the oscillator to a predetermined value and maintaining it at a level at which frequency stability of around 1 ppb can be obtained. Although the OCXO has high frequency stability, because of the development of micro-electro-mechanical system technology with characteristics of low cost, low power consumption, integration, and multi-function, the OCXO has fewer applications due to its shortcomings, such as high power consumption and integration difficulties [9]. Because of its low power consumption and easy integration [10], the TCXO has become the mainstream choice for multi-device system clocks. The stability of the TCXO is relatively low and has a significant influence on the synchronization effect. In recent years, research has improved the algorithm to reduce the effect of the TCXO stability on the synchronization effect [11,12,13,14], but the effect is limited. If the frequency stability of the TCXO can be further improved, a better synchronization effect can be obtained. In addition to the higher frequency stability of the TCXO indoor positioning [15], the control of unmanned aerial vehicles (UAVs) [16] and high-precision time measurement [17] show good application potential.

Two methods exist to implement a TCXO, namely the analog method [18] and the digital method [19]. The analog method achieves temperature compensation by constructing a peripheral network complementary to the temperature coefficient of the crystal oscillator. The digital method first reads the current temperature using the temperature sensor, and then adjusts the crystal oscillator according to the stored data. Generally, digital methods can achieve higher frequency stability and boast a wider temperature compensation interval.

The performance of digital methods can be increased by increasing the measurement density of compensation points, and improving the accuracy of temperature sensors and crystal oscillator control. However, constant changes of the ambient temperature will inevitably cause thermal hysteresis of the crystal oscillator. Thermal hysteresis of the crystal oscillator means that the output frequency of the crystal oscillator cannot be completely duplicated during the temperature cycle. This is attributed to the orientation and stress structure of the quartz crystal, which is generally several hundred ppb [20].

The frequency stability of the TCXO designed in [21] and [22] reached ±1 ppm and ±0.5 ppm, respectively; however, these studies only performed regular compensation for the crystal oscillator and did not consider the thermal hysteresis effect. If thermal hysteresis were taken into consideration, more errors would be introduced and TCXO frequency stability could be further improved. Without relevant discussion, further improvements would be possibly halted.

Regarding the reduction in the frequency deviation caused by the thermal hysteresis effect of the crystal oscillator, the authors in [23] reduced the thermal hysteresis effect of the crystal by changing the stress structure of the crystal, which completely integrates the crystal oscillator and the complementary metal oxide semiconductor (CMOS) substrate. Because of the use of low-temperature vacuum packaging technology, a hysteresis error of −17 to 70 °C was reduced to 66 ppb, which in turn increased the stability of the TCXO to ±190 ppb. However, this method is complicated. In terms of process and budget, the authors in [24] reduced the effect of thermal hysteresis by selecting a specially oriented crystal oscillator. The thermal hysteresis effect of the third overtone SC-cut crystal oscillator was only about 10 ppb, and the frequency stability of the final designed TCXO reached about ±30 ppb. The authors in [25] show that, through strict process control, the hysteresis error of the SC-cut crystal oscillator can be reduced to several ppb, which can further improve the frequency stability of the TCXO. In the crystal cutting process, the SC-cut crystal needs to rotate twice, whereas the AT-cut crystal only needs to rotate once; thus, the cost of the SC-cut crystal is considerably higher than that of the AT-cut crystal. Although SC-cut crystals have better temperature stability and aging characteristics, cheaper AT-cut crystals are more widely used. Therefore, the use of a cheaper AT-cut crystal to achieve higher frequency stability while reducing hysteresis error would be highly significant.

This article proposes a low-cost, highly applicable method that can compensate for the thermal hysteresis of the crystal oscillator in the TCXO, reduce the influence of the thermal hysteresis of the crystal oscillator on the TCXO frequency, and improve the performance of the TCXO frequency stability, that is, based on the current temperature information and the history information of temperature changes, compensating the crystal oscillator for thermal hysteresis while performing temperature frequency compensation. In addition, a specially designed TCXO is implemented that reduces the compensated hysteresis error of −40 to 80 °C from about 700 ppb to about 150 ppb.

## 2. Thermal Hysteresis of Crystal Oscillator

**Figure 1:**
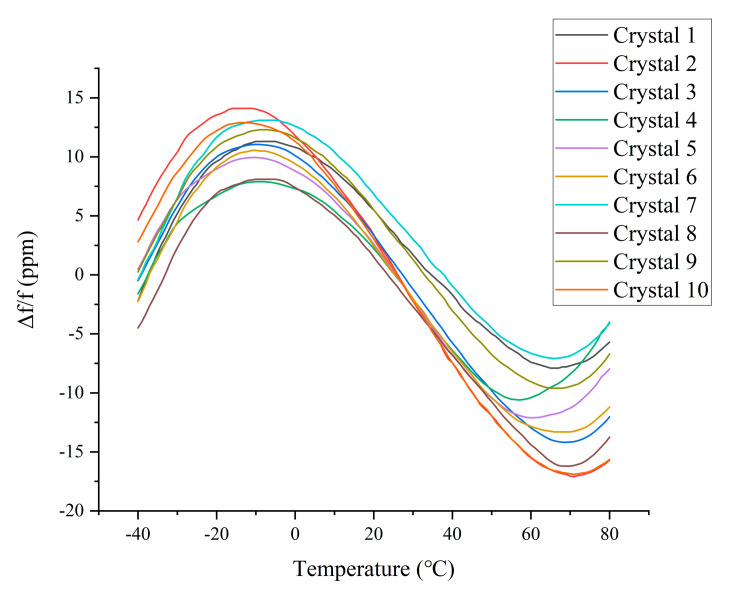
Crystal frequency–temperature diagram.

The crystal oscillator used in the TCXO designed for this study is an AT-cut crystal oscillator. The output frequency change curve of this crystal oscillator with temperature is an approximate cubic curve, presenting a lying “S” shape, with two inflection points at about −10 and 65 °C. The frequency stability of the AT-cut quartz crystal oscillator used in this article is 20 ppm, and temperature is the main factor affecting its frequency stability [20]. For comparison, this study used 10 AT-cut crystal oscillators with a nominal frequency of 10 MHz. Their temperature–frequency curves are shown in Figure 1, where f = 10 MHz.

**Figure 2:**
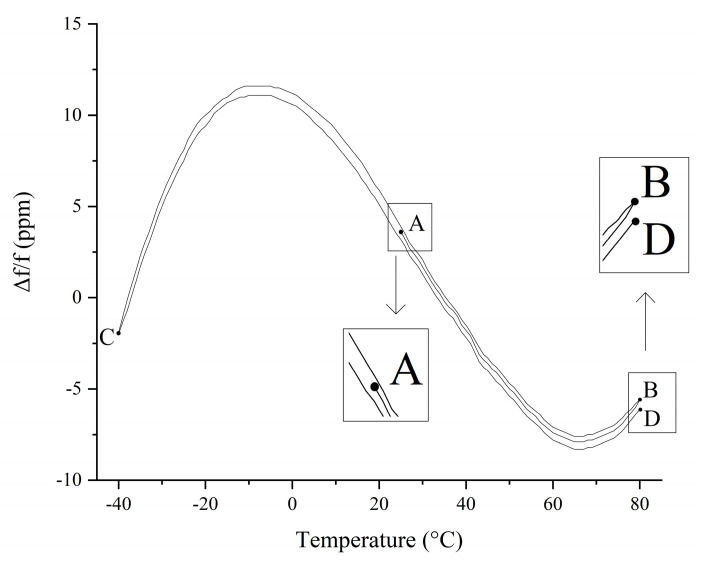
Frequency–temperature curve of crystal 1 during crystal temperature cycling.

However, the crystal frequency change curve with temperature does not repeat identically when the temperature cycle changes [26]. Figure 2 shows the frequency–temperature curve of crystal 1 in the thermal cycle, where f = 10 MHz. The thermal cycling process of A→B→C→D first rises from 25 to 80 °C (AB segment), then drops from 80 to −40 °C (BC segment), and finally rises from −40 to 80 °C (CD segment). Figure 2 shows that, in this temperature cycle, the BC and CD segments of the crystal oscillator frequency–temperature curve are obviously different. This phenomenon is the thermal hysteresis of the crystal. After multiple measurements under the temperature cycle from −40 to 80 °C, it was found that both the rising and dropping edges of the temperature curve have good repeatability throughout the temperature cycle. The same phenomenon was measured for the other nine crystals

This phenomenon is the thermal hysteresis of the crystal oscillator, which is caused by the stress inside the crystal. Temperature cycling can produce changes in the stress on the resonator plate. The stress on the resonator includes mounting stress (through force–frequency effects [27,28] and bending effects [29]), bonding stress [30], and electrode stress [31]. These stress changes on the resonant plate produce frequency shifts. The stress structure of different tangential crystals is different, so the degree and characteristics of the thermal hysteresis effect of the AT-cut, BT-cut, and SC-cut crystals are also different [32,33,34].

**Figure 3:**
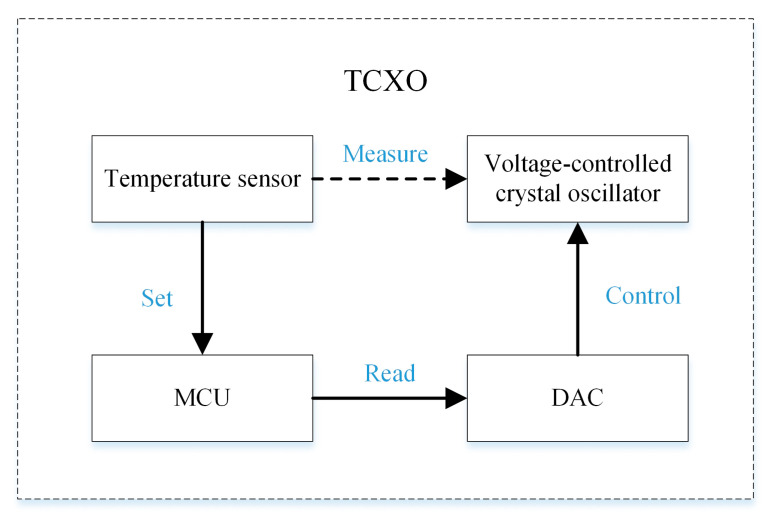
Temperature-compensated X’tal (crystal) oscillator (TCXO) structure block diagram. Note: Micro Controller Unit (MCU); Digital to Analog Converter (DAC).

**Figure 4:**
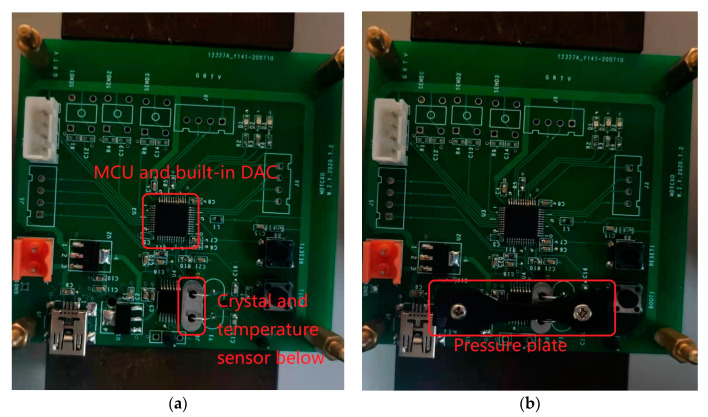
(**a**) TCXO physical diagram before installing the pressure plate; (**b**) TCXO physical diagram after installing the pressure plate.

These crystal oscillators were used as clock sources of TCXOs to better demonstrate their thermal hysteresis. The structure diagram of the TCXO designed for this study is shown in Figure 3, and the physical diagram is shown in Figure 4. The pressure plate in the picture enables closer contact between the crystal and the temperature sensor. The methods used to install the crystal and temperature sensor and measure the temperature are described in more detail below.

In this TCXO, the Micro Controller Unit (MCU) communicates with the temperature sensor through the I2C bus, and sets the Digital to Analog Converter (DAC) voltage according to the measured temperature to adjust the output frequency of the TCXO. Before using the TCXO, the compensation value of the DAC is first measured at different temperatures. When measuring the compensation value, the TCXO is placed in the incubator. After the temperature in the incubator stabilizes for 5 min, the DAC voltage is continuously adjusted until the TCXO output frequency is equal to the target frequency (10 MHz in this article), and measurements are taken every 0.5 °C between −40 and 80 °C. Note that, at this time, the temperature read by the temperature sensor on the TCXO prevails rather than the temperature displayed on the incubator, because the temperature displayed on the incubator is measured by the temperature sensor on the inner wall of the incubator, which does not represent the temperature of the TCXO. Moreover, when the TCXO is working, the frequency is adjusted based on the temperature sensor reading on the TCXO; thus, when measuring the DAC compensation value, the TCXO temperature sensor reading should prevail. After the compensation value is measured, the measurement result is entered into a compensation table that corresponds to the temperature and DAC voltage, and writes it into the MCU. When the TCXO is working, the MCU reads the temperature sensor value each second, and obtains the corresponding compensation value from the previously measured DAC compensation value table according to the temperature data measured by the temperature sensor. It then sets this compensation value to the DAC to adjust the TCXO output frequency.

Under normal circumstances, after completing a compensation value measurement, the TCXO cannot achieve the desired frequency stability, and error correction is required, that is, the compensation table is first written into the MCU, and the TCXO works at different temperatures and its frequency is measured. If the frequency is too far from the target frequency, the DAC compensation value should be adjusted and updated in the compensation table in the MCU. After multiple corrections, the frequency stability of the TCXO is improved. For large-scale industrial TCXOs, the compensation values of several key temperature nodes are often measured and then interpolated to obtain a complete compensation table. In contrast, the method used in this article is less efficient but has better compensation results. Because the main aim of this article was to study the thermal hysteresis effect of a TCXO, a TCXO with higher frequency stability was required to clearly observe the effect; hence, the presented method was adopted.

**Figure 5:**
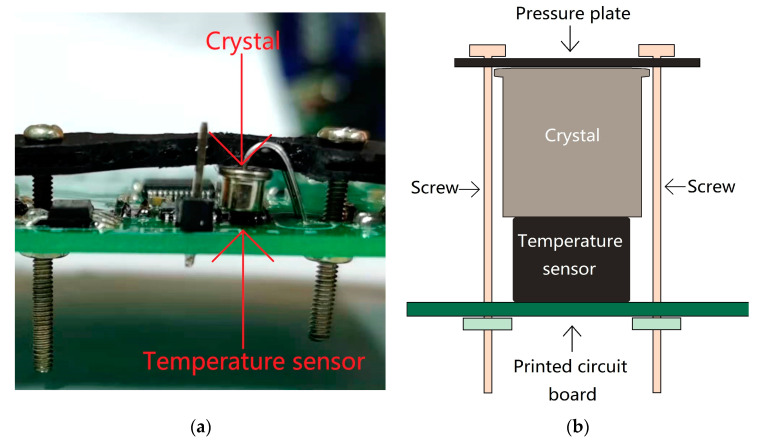
(**a**) TCXO physical diagram (side view); (**b**) temperature measurement diagram.

The temperature sensor used in the TCXO was an MCP9808 of the Microchip Technology Inc. (Chandler, AZ, USA) The measurement accuracy of this temperature sensor is ±0.25 °C (typical value) from −40 to +125 °C. The temperature measurement method in this article is shown in Figure 5. In the picture, thermal grease is applied between the temperature sensor and the crystal, which increases the thermal coupling efficiency between the them. The thermal hysteresis effect measured in this article is the thermal hysteresis effect of the entire TCXO system, including the thermal hysteresis of the crystal oscillator and other thermal components, which is generally greater than the “true hysteresis” of the crystal oscillator [35]. All of the temperature data involved in this article are measured by the temperature sensor on the TCXO. The temperature data are read by the MCU through the I2C bus, and then sent to the computer through the serial port and recorded.

According to the method mentioned above, a TCXO made of ten crystal samples as a clock source is measured by compensation meter. After sequentially debugging the compensation points with poor compensation effect, the frequency stability of the TCXO without considering thermal hysteresis was ±150 ppb.

**Figure 6:**
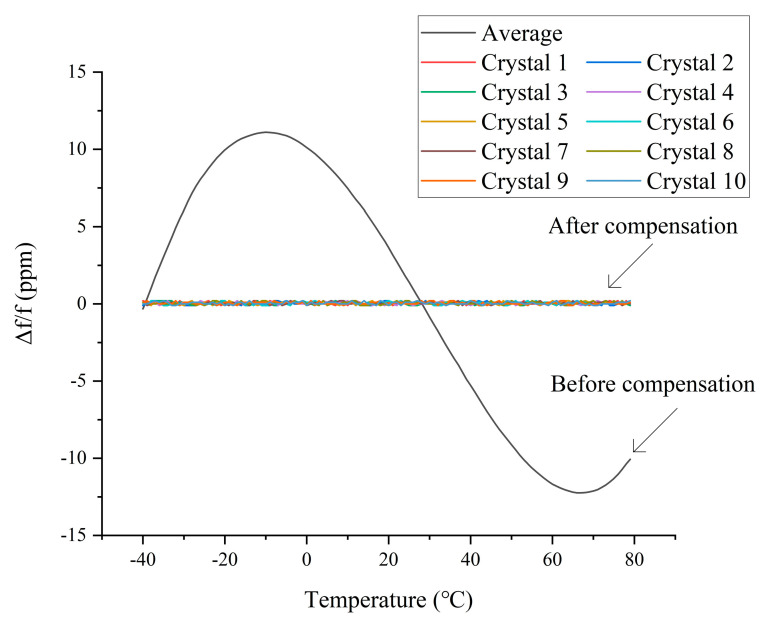
Comparison chart before and after compensation.

The compensation result is shown in Figure 6, where the curve marked “Average” is the average curve of the ten curves in Figure 1. This is to make Figure 6 easier to read. It can be seen from Figure 6 that after temperature compensation the frequency stability of the crystal was significantly improved.

After digital compensation, we conducted a thermal cycling experiment on the TCXO with crystal 1 as the clock source; the temperature cycle test was conducted and the test result is shown in Figure 4. The test process was to first raise the temperature from 25 to 80 °C (AB segment), then drop it from 80 to −40 °C (BC segment), then raise it from −40 to 70 °C (CD segment), and finally drop it from 70 to −40 °C (DE segment).

**Figure 7:**
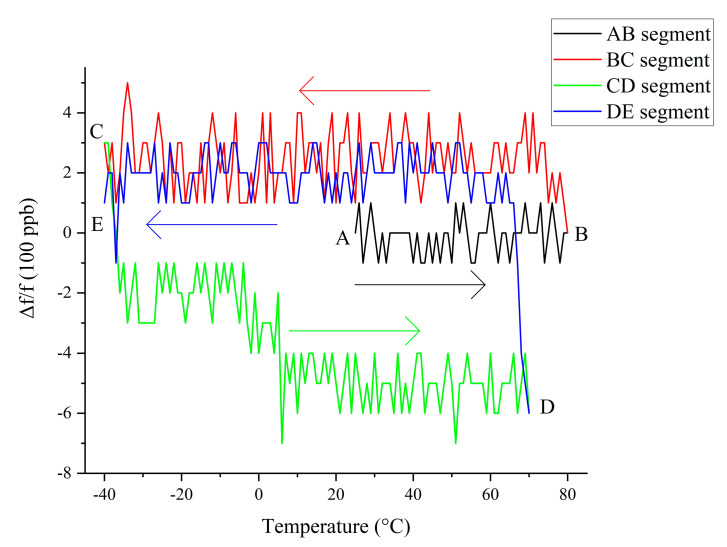
Original thermal hysteresis curve.

Through the digital compensation of the crystal oscillator, the impression of the crystal oscillator’s thermal hysteresis on the frequency stability of the TCXO can be more clearly observed in Figure 7. However, the conventional TCXO does not perform temperature cycle testing because, when its frequency stability is a few ppm, the thermal hysteresis error of the crystal is generally several hundred ppb, and such a small error is not obvious when it is added to a few ppm [20]. To improve the frequency stability of these TCXOs, it is more effective to improve the accuracy of thermal compensation than to compensate for hysteresis errors.

In Figure 7, the temperature drop curve (segment BC, segment DE) can be approximated as a straight line after compensation, whereas the temperature rise curve (segment CD) has obvious steps. This is because the compensation point in this experiment is measured during the temperature drop process, so the temperature drop curve compensation effect performs better than the temperature rise curve.

**Figure 8:**
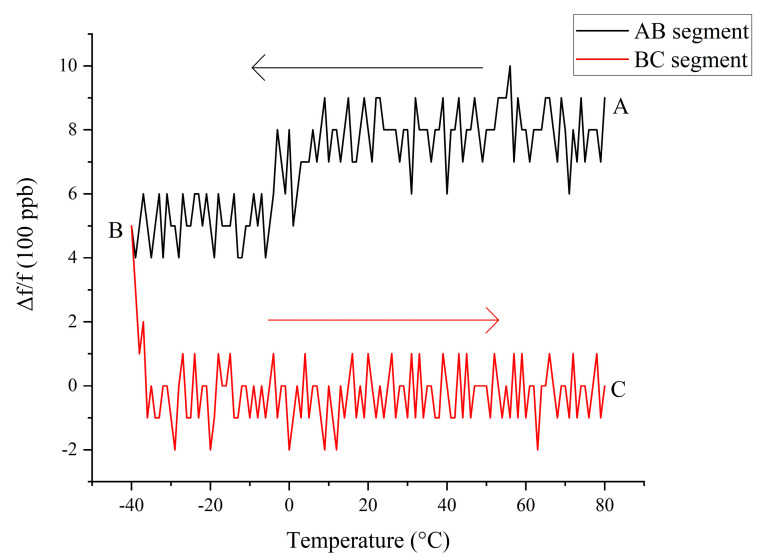
Hysteresis test diagram of compensation point measurement during the temperature rise process.

Figure 8 shows the result of measuring compensation points during the rising process. The test process was to first drop the temperature from 80 to −40 °C (AB segment), and then raise it from −40 to 80 °C (BC segment). Figure 8 shows the opposite compensation result, that is, the temperature rise curve (segment BC) can be approximated as a straight line after compensation, and there are obvious steps in the temperature drop curve (segment AB).

**Figure 9:**
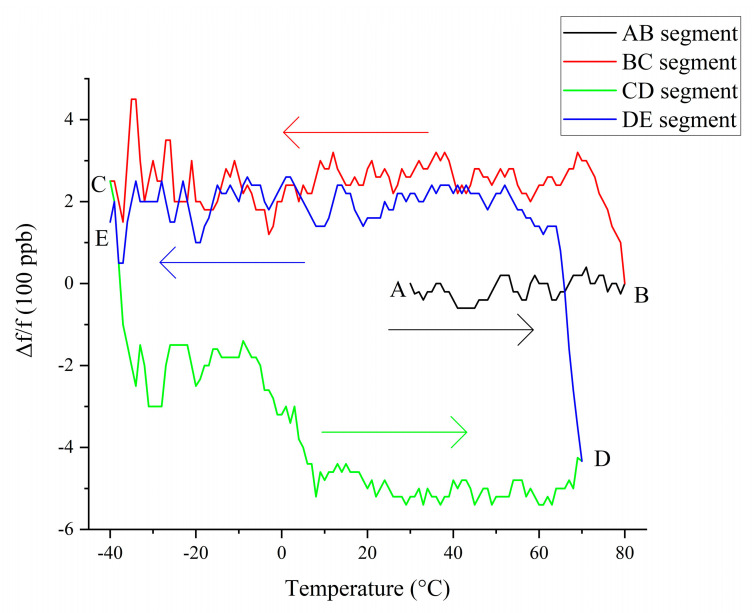
Thermal hysteresis curve after smoothing.

For a clearer observation, a smoothing process was performed on Figure 7, thus yielding Figure 9. The AB, BC, and DE segments in Figure 9 can be approximated as straight lines parallel to the horizontal axis. Although there is a step in the CD segment, which cannot be approximated as a straight line parallel to the horizontal axis, it can be regarded as two straight lines parallel to the horizontal axis with 10 °C as the boundary.

Comparing and observing Figure 7 and Figure 9, we can clearly see that each output frequency curve of the TCXO in Figure 7 can be approximately regarded as a small-range jitter centered on the approximate straight line in Figure 9. This fluctuation is caused by the error of the temperature sensor and the DAC, which means the TCXO is unable to completely achieve accurate compensation. This jitter is the frequency instability mentioned in the TCXO that occurs when the effect of thermal hysteresis is not taken into consideration. In this experiment, the amplitude of this jitter is ±150 ppb.

Observing Figure 9, we can see that, at each change in the direction of temperature change (points B, C, and D), the output frequency of the TCXO changes significantly. When the operating temperature of the TCXO is greater than 10 °C, the frequency curve before and after the change can be approximated as several parallel lines. When the temperature changes direction, the TCXO output frequency curve approximates a new center frequency f_0_, which is called the hysteresis frequency. Therefore, another evaluation criterion for the frequency stability of the TCXO, i.e., hysteresis error, can be introduced. The temperature-compensated crystal oscillator can measure multiple different frequency–temperature curves during the temperature rise and temperature drop processes, in addition to each curve’s jitters near its own hysteresis frequency f_0_. The hysteresis error is used to measure the output frequency offset during the temperature cycle; its value is $f_{0max}-f_{0min}/f$, where f represents the target compensation frequency of the TCXO. In this experiment, f = 10 MHz. When the working temperature of the TCXO is less than 10 °C, there is no longer a simple transition between the hysteresis curves, and the shape of the curve changes slightly.

According to multiple measurements and verification, it was found that the thermal hysteresis of the crystal oscillator has the following characteristics:

1. Thermal hysteresis only occurs when the direction of the temperature variation curve changes, and unidirectional temperature variation does not produce thermal hysteresis.
2. The hysteresis frequency is related to the temperature of the crystal when the direction of temperature changes [36], and has little connection with the direction of temperature change.
3. The hysteresis frequency has no relationship with the speed of the temperature change [35]. If the temperature changes too fast, the temperature lag between the temperature sensor and the crystal causes frequency compensation errors and frequency offset.
4. A small range of temperature changes does not cause thermal hysteresis. The samples tested in the experiment do not produce thermal hysteresis when the temperature change range is not greater than ±1 °C.

**Figure 10:**
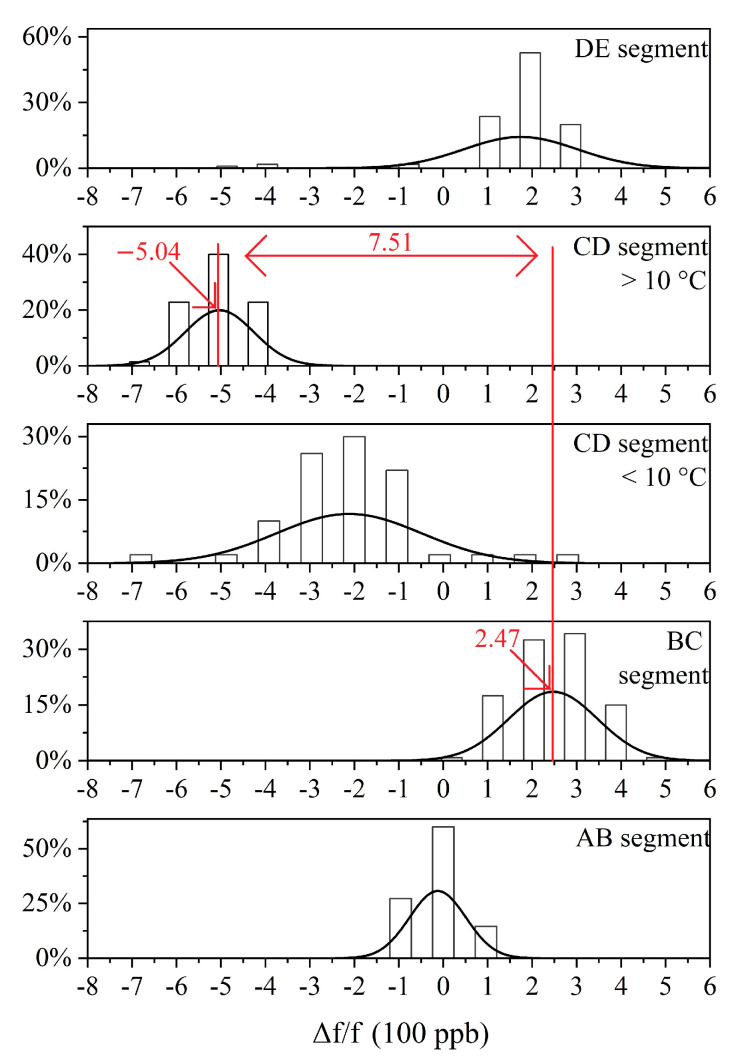
Frequency distribution diagram before hysteresis compensation.

Figure 10 shows the frequency distribution diagram of the curves in Figure 7. Observing Figure 7, it is obvious that the CD segment is divided into two parts, i.e., greater than 10 °C and less than 10 °C. Each part can be approximately regarded as a straight line parallel to the abscissa. Therefore, in Figure 10, we also divide the CD segment into two parts, i.e., greater than 10 °C and less than 10 °C. In Figure 10, the frequency distribution diagram of each frequency curve segment presents an approximate normal distribution. The frequency of the midpoint of the normal distribution is the hysteresis frequency f_0_ mentioned in the previous section. As can be seen, the hysteresis frequency changes each time the direction of the temperature changes. In addition to the points falling on the hysteresis frequency and the points around the hysteresis frequency, some points that deviate from the hysteresis frequency can also be observed. These points are often small in number, and are measured during the thermal hysteresis of the crystal oscillator, that is, just after changes in the direction of the temperature change. Observing Figure 7 and Figure 10, we can see that the hysteresis error of the experimental TCXO is 751 ppb. Thermal hysteresis is the overall phenomenon of the entire TCXO system. In addition to crystals, other components of the circuit also introduce different levels of thermal hysteresis [37].

In this study, crystals 2–10 were also used as the clock source to make TCXOs, and the tests outlined above were repeated. The thermal hysteresis of these TCXOs was similar to that of the TCXO with crystal 1 as the clock source, but the degree of hysteresis was different. The test results are shown in Table 1. As shown in Table 1, TCXOs with different crystals as clock sources have different degrees of thermal hysteresis error. The average of these thermal hysteresis errors is about 700 ppb.

## 3. Compensation for Hysteresis

The frequency stability of the TCXO designed for this study is ±150 ppb, and the hysteresis error is about 700 ppb. The effect of hysteresis error on the overall frequency stability of the crystal cannot be ignored, and the thermal hysteresis phenomenon must be compensated for.

Due to the microprocessor embedded in the TCXO, it is possible to compensate for thermal hysteresis. We can save the temperature data obtained by the temperature sensor, traverse the historical temperature data each time the compensation value is set, analyze whether the crystal has a temperature change that is sufficient to cause thermal hysteresis, and set the corresponding compensation according to the analysis result.

This study used a fixed-length doubly linked list to store temperature historical data. Each node of the linked list holds two values, namely temperature t and the temperature duration T. The unit of temperature t is degrees Celsius. In this experiment, the processor reads the temperature sensor data each second, so the unit of time T is seconds. Because all of the data in the linked list must be traversed each time the thermal hysteresis is judged, a linked list that is too long reduces the judgment efficiency. However, if the length of the linked list is reduced blindly for efficiency, the temperature history information in the linked list is not valuable enough, thus making it impossible to accurately determine whether thermal hysteresis occurs. Therefore, it is necessary to comprehensively value the trade-offs involved in selecting the proper length of data.

**Figure 11:**
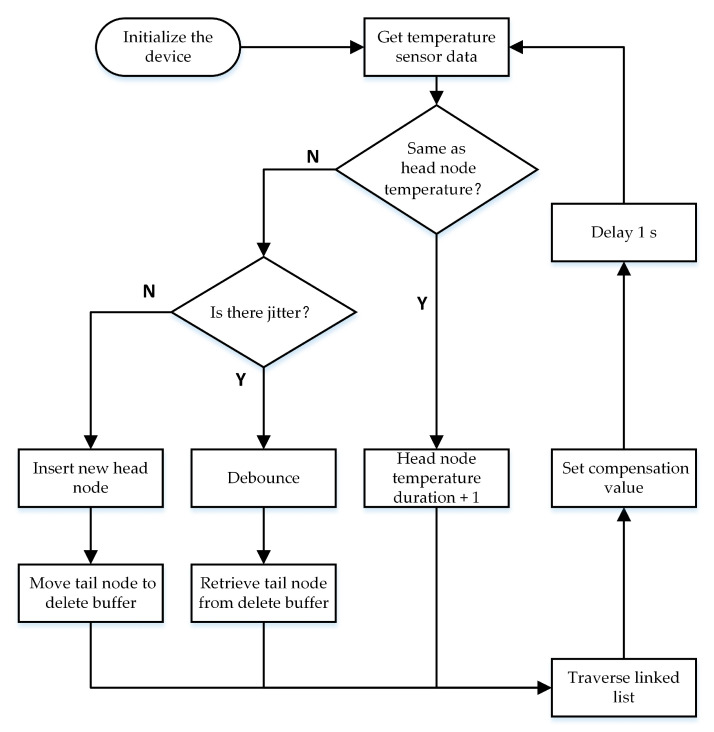
Flow chart of thermal hysteresis compensation.

The processing steps of the TCXO are shown in Figure 11. When the TCXO is working normally, the temperature data of the temperature sensor is read once per second, and the temperature data read from the temperature sensor is then compared with the temperature data t stored in the head node of the linked list; if they are the same, the temperature duration T stored in the head node of the linked list is incremented by 1. If they are different, the sensor data is compared with the temperature data t stored in the second node of the linked list. If they are the same, it means that there is a small temperature jitter, which may be caused by the measurement error of the temperature sensor, and the small temperature jitter does not cause the crystal thermal hysteresis. Experimental measurements show that this small temperature jitter is very common. If these small temperature jitters are not addressed, the list contains useless data, making it impossible to accurately judge the hysteresis. To avoid this situation, when a small temperature jitter is observed, that is, the temperature data read by the temperature sensor is the same as the temperature data stored in the second node of the linked list, the useless recorded data should be deleted due to the small temperature jitter. When a small temperature jitter is detected, the temperature duration T of the head node is added to the temperature duration T of the second node in the list, and the head node is then deleted. As a result of deleting the head node, the length of the linked list changes. Therefore, we must establish a deletion buffer to store the tail node that was deleted. In this way, after deleting the head node, a tail node can be taken from the buffer and inserted into the tail of the list to maintain the length of the linked list. When the temperature data read by the temperature sensor is different from the temperature data stored in the head node of the linked list and there is no jitter, a new head node is inserted at the head of the linked list, and the temperature t and duration T are stored. Then, the tail node is deleted and the tail node is stored in the delete buffer.

After completing the update of the linked list, it should be traversed and the algorithm judges whether hysteresis compensation is needed by checking the temperature change history. It is necessary to measure the compensation table for hysteresis at different temperatures and check the table according to the temperature when hysteresis occurs during compensation in advance. After testing, the hysteresis frequency of the TCXO in this experiment changes by 10 to 15 °C and its hysteresis frequency changes by 1 Hz, which differs slightly for different crystals. This experiment can achieve a minimum frequency adjustment accuracy of 1 Hz, so the new compensation table is measured each 10 °C. When the TCXO operating temperature is greater than 10 °C (see Figure 9), different hysteresis curves can be approximately regarded as parallel to each other. Therefore, if the crystal operating temperature is greater than 10 °C, the method of adding a fixed offset value to the original compensation value can be used, although this method has a poor compensation effect.

In addition, when the temperature changes too quickly, due to the different thermal conductivity of the temperature sensor and the crystal surface material, there is a certain thermal lag between the two. The temperature change rate can also be estimated based on the temperature and duration information in the linked list, and compensated for. Physically, it is also possible to increase the heat transfer efficiency between the two by adding heat dissipating silicone grease or filling with dry nitrogen to reduce the relative temperature difference. This is not the focus of this article and will not be discussed in detail.

## 4. Compensation Results

**Figure 12:**
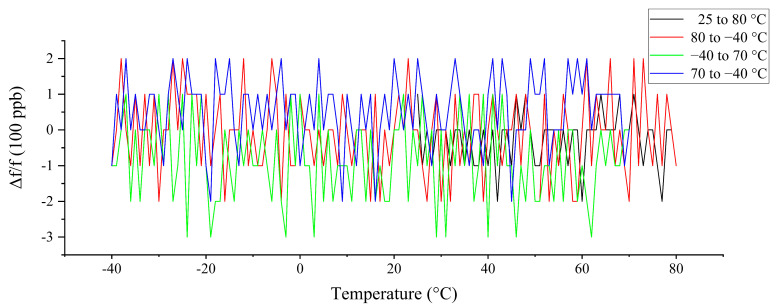
Frequency–temperature curve after thermal hysteresis compensation (−40 to 80 °C).

**Figure 13:**
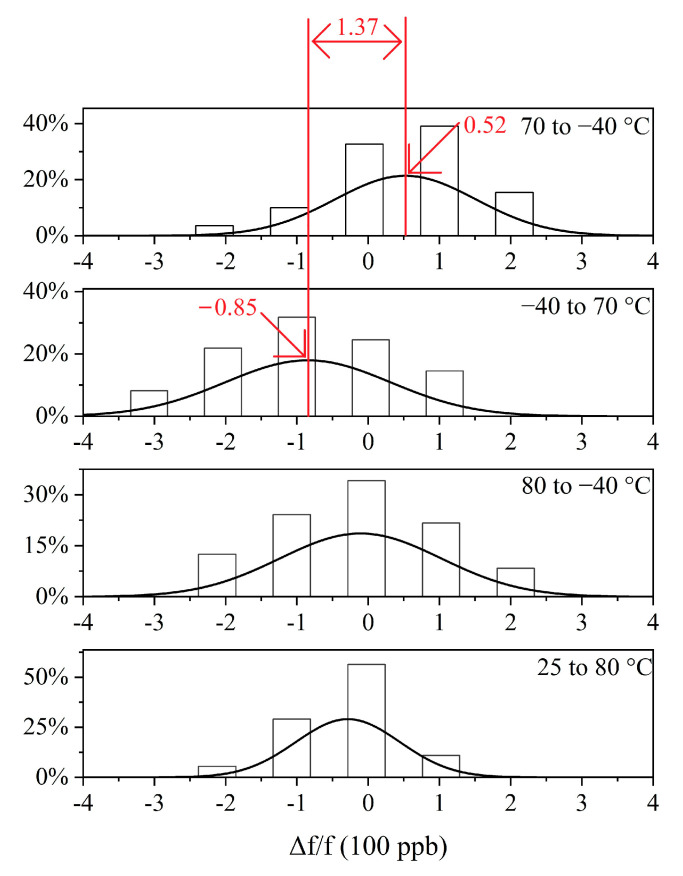
Frequency distribution diagram after hysteresis compensation.

According to the compensation method proposed in the previous section, the compensation code of the TCXO is rewritten. Because there is a microprocessor in the TCXO, there is no need to redesign the hardware. The compensation result of the TCXO driven by crystal 1 is shown in Figure 12. The working temperature cycle process of the crystal in Figure 12 is as follows: first raising the temperature from 25 to 80 °C, then dropping it from 80 to −40 °C, then raising it from −40 to 70 °C, and finally dropping it from 70 to −40 °C. Figure 13 shows the frequency distribution diagram of the four curves in Figure 12.

Observing and comparing Figure 7 and Figure 12, it can be seen that, after the thermal hysteresis compensation shown in Figure 7, the separated curve sections are obviously close together in Figure 12. In addition, due to the independent compensation points measured during both the heating and cooling processes, the step phenomenon in the CD section of Figure 7 that is less than 10 °C does not appear in Figure 9. Observing Figure 12 and Figure 13, and comparing Figure 7 and Figure 10, it can be seen that the hysteresis stability of the crystal oscillator was significantly improved after compensation, and the hysteresis error of −40 to 80 °C was reduced from 752 to 137 ppb. After hysteresis compensation, the frequency stability of the crystal drops slightly, from ±150 to ±200 ppb.

The TCXOs with crystals Nos. 2–10 as the clock source were also subjected to the same compensation and testing. After compensation, the measured hysteresis error is shown in Table 2. Table 2 shows that after all 10 samples were compensated for thermal hysteresis, the hysteresis error was reduced to about 150 ppb. This study also tried to adjust the compensation value to improve the accuracy of thermal hysteresis compensation; however, due to the DAC’s temperature sensor accuracy, compensation of around 150 ppb was the best that could be achieved.

As shown in Table 1 and Table 2, for this study we made TCXOs for ten different crystal samples, using these as clock sources, and performed thermal hysteresis compensation on them. For each sample, the thermal hysteresis was reduced to varying degrees after compensation, and the average hysteresis error of the final 10 samples after compensation was about 150 ppb. This shows that the thermal hysteresis compensation method proposed in this article can reduce the hysteresis error of the TCXO.

## 5. Conclusions

In this study, the thermal hysteresis phenomenon of crystals was analyzed. A corresponding compensation model was constructed, a compensation algorithm for the thermal hysteresis phenomenon was designed, and the compensation of the thermal hysteresis for the crystal oscillator was realized. The thermal hysteresis error of −40 to 80 °C was reduced from 700 to 150 ppb. Thus, this study addressed a problem of the traditional TCXO, namely that it cannot compensate for the frequency offset caused by the thermal hysteresis of the crystal oscillator in scenarios with a large temperature variation range. Hence, the frequency stability of the crystal oscillator was significantly improved.

The method proposed in this article improves the stability of TCXOs with little additional power cost, and can be applied to multi-sensor networks that must be synchronized to increase their synchronization accuracy [38,39]. This method can also be applied to power-sensitive sensor network systems, such as the one investigated in the previous research of our experimental team [40], in which we used the GPS signal to tune the crystal oscillator for a high frequency stability. Using the TCXO designed for this study to replace the GPS module could reduce system power consumption while maintaining synchronization accuracy.

## Figures and Tables

**Table 1:** Maximum thermal hysteresis error of each TCXO before thermal hysteresis compensation.

Crystal Number	Maximum Thermal Hysteresis Error (ppb)
1	752
2	638
3	529
4	916
5	594
6	677
7	821
8	703
9	533
10	729
Average = 700, δ = 120	

**Table 2:** Maximum thermal hysteresis error of each TCXO after thermal hysteresis compensation.

Crystal Number	Maximum Thermal Hysteresis Error (ppb)
1	137
2	156
3	168
4	170
5	131
6	146
7	161
8	164
9	142
10	169
Average = 150, δ = 14	
